# Sugar-Modified Poly(propylene imine) Dendrimers Stimulate the NF-κB Pathway in a Myeloid Cell Line

**DOI:** 10.1007/s11095-016-2049-3

**Published:** 2016-10-20

**Authors:** Izabela Jatczak-Pawlik, Michal Gorzkiewicz, Maciej Studzian, Dietmar Appelhans, Brigitte Voit, Lukasz Pulaski, Barbara Klajnert-Maculewicz

**Affiliations:** 1 Department of General Biophysics, Faculty of Biology and Environmental Protection, University of Lodz, 141/143 Pomorska St., 90-236 Lodz, Poland; 2 Department of Molecular Biophysics, Faculty of Biology and Environmental Protection, University of Lodz, 141/143 Pomorska St., 90-236 Lodz, Poland; 3 Leibniz Institute of Polymer Research Dresden, Hohe Str. 6, 01069 Dresden, Germany; 4 Laboratory of Transcriptional Regulation, Institute of Medical Biology PAS, 106 Lodowa St., 93-232 Lodz, Poland

**Keywords:** glycodendrimers, immunomodulation, monocytes, NF-κB, PPI dendrimers

## Abstract

**Purpose:**

Fourth-generation poly(propylene imine) dendrimers fully surface-modified by maltose (dense shell, PPI-m DS) were shown to be biocompatible in cellular models, which is important for their application in drug delivery. We decided to verify also their inherent bioactivity, including immunomodulatory activity, for potential clinical applications. We tested their effects on the THP-1 monocytic cell line model of innate immunity effectors.

**Methods:**

To estimate the cytotoxicity of dendrimers the reasazurin assay was performed. The expression level of NF-κB targets: *IGFBP3*, *TNFAIP3* and *TNF* was determined by quantitative real-time RT-PCR. Measurement of NF-κB p65 translocation from cytoplasm to nucleus was conducted with a high-content screening platform and binding of NF-κB to a consensus DNA probe was determined by electrophoretic mobility shift assay. The cytokine assay was performed to measure protein concentration of TNFalpha and IL-4.

**Results:**

We found that PPI-m DS did not impact THP-1 viability and growth even at high concentrations (up to 100 μM). They also did not induce expression of genes for important signaling pathways: Jak/STAT, Keap1/Nrf2 and ER stress. However, high concentrations of 4th generation PPI-m DS (25–100 μM), but not their 3rd generation counterparts, induced nuclear translocation of p65 NF-κB protein and its DNA-binding activity, leading to NF-κB-dependent increased expression of mRNA for NF-κB targets: *IGFBP3*, *TNFAIP3* and *TNF*. However, no increase in pro-inflammatory cytokine secretion was detected.

**Conclusion:**

We conclude that maltose-modified PPI dendrimers of specific size could exert a modest immunomodulatory effect, which may be advantageous in clinical applications (e.g. adjuvant effect in anti-cancer vaccines).

## Introduction

Since the early 1980s, dendrimers have been comprehensively studied for various medical applications (1). These highly branched macromolecules offer many advantages when compared to other chemical polymers with different architectural forms (2). Thanks to the development of precise methods of synthesis it became possible to obtain homogeneous compounds with specified chemical features and well-defined structure. This includes a central core with radially attached polymeric branches and peripheral functional groups (3). All those elements affect the size and shape of the dendrimer, at the same time providing a number of unique properties (4). Dendrimers are used both in drug delivery and as bona fide bioactive compounds, however it is important to distinguish the chemical and biochemical characteristics that make them suitable for these goals.

The biological features of dendrimers, such as high solubility and biopermeability, are affected by the character of highly-reactive terminal moieties (5). Unfortunately, in some polyamine dendrimer types, e.g. poly(propylene imine) (PPI) dendrimers, the properties of the dendritic scaffold are too harsh, making them bio-incompatible for direct application. Therefore, intensive research is being performed on properties of dendrimers with diverse end group modifications. Because of non-specific interactions with negatively-charged biological membranes, cationic dendritic polymers, such as poly(amidoamine) (PAMAM) or poly(propylene imine) (PPI) dendrimers, are more hemolytic and cytotoxic (6,7) in comparison to neutral and anionic macromolecules (8). A positive surface charge significantly limits the medical applicability of this type of dendrimers. Therefore, it is important to find the right balance between toxic side effects and unique properties allowing the therapeutic application of dendrimers. The reduction of toxicity of positively charged macromolecules is possible through chemical modifications of terminal groups. For this purpose, poly(ethylene glycol) chains have been most commonly studied (9), as well as fatty acids (7), and acetyl or glycidol groups (6). In addition, certain surface modifications have been found to improve the targeting potential, biocompatibility and biodistribution of dendrimers (10,11).

In order to develop efficient drug delivery system, scientific interest turned to glycodendrimers (7,12,13). Modification with sugar moieties has been found to significantly lower the cytotoxic activity of dendrimers, as well as prolong their blood half-life, improve biocompatibility and loading capacity (12–14). Carbohydrates may also enhance the molecular recognition potential of glycodendrimers thanks to the specific interactions with lectin receptors overexpressed on various types of cancer cells (15).

Maltose-coated PPI dendrimers have shown a very good combination of biocompatibility and bioactivity (12,13). While fully surface-modified PPI-m glycodendrimers are unsuitable as drug delivery vehicles for anionic drug molecules (16), they have unique bioactivities that are just starting to be characterised. Our research concentrates on validating potential applications of maltose-modified glycodendrimers due to their specific interactions with biological systems, including immune recognition pathways. Immunomodulation is a very important goal in nanopharmacology, because specific and targeted activation or inhibition of immune cell proliferation, antibody secretion or cytokine release would be preferable to currently available broad-target chemical immunomodulators which have severe side effects (17). Since oligosaccharide end groups are very important in immune recognition, we set out to test the potential immunomodulatory potency of PPI-m glycodendrimers, as exemplified by signaling pathways in the THP-1 cell line as monocyte model.

## Materials and Methods

### Material

#### Dendrimers

**Scheme 1:**
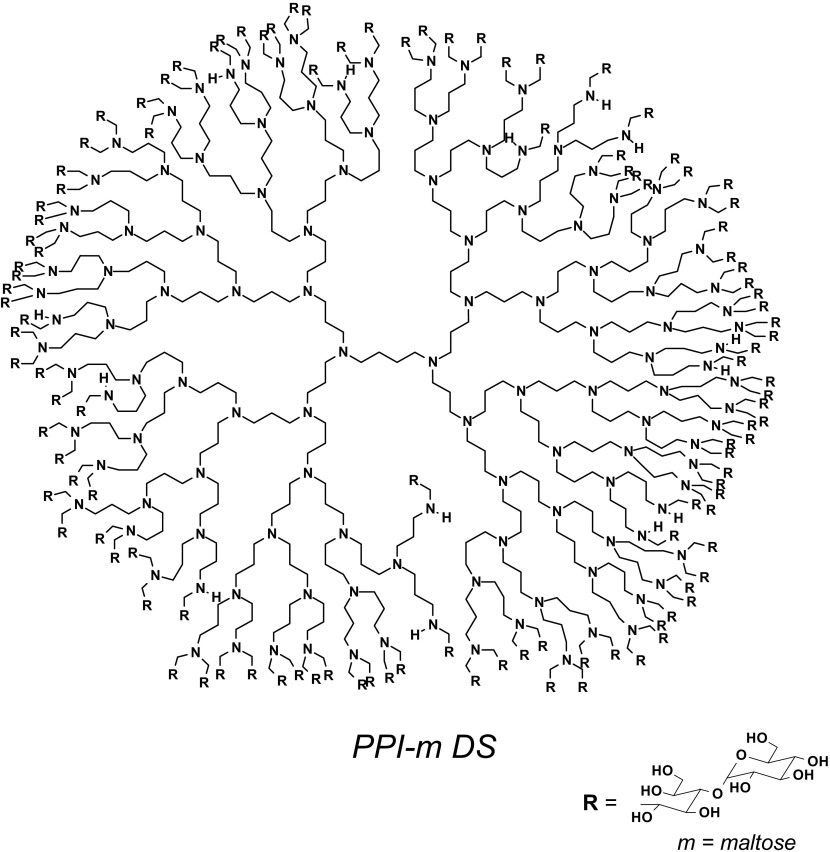
The structure of PPI dendrimer of the 4th generation modified with maltose

Poly(propylene imine) dendrimer of the 4th generation with primary amino surface groups was obtained from Symo-Chem (Eindhoven; Netherlands) and modified with maltose to 90% extent (dense shell glycodendrimers; PPI-m DS G3, MW 24,397 g/mol or PPI-m DS G4, MW 44,500 g/mol) (Scheme 1). Dendrimers were synthesized, purified and characterized as previously described (18). The molecular weight of the PPI glycodendrimer was determined by an established ^1^H NMR approach (19).

### Methods

#### Cell Culture

THP-1 (acute monocytic leukemia) human cell line was purchased from ATCC (Manassas, VA, USA) and maintained under standard conditions in RPMI-1640 Medium (ThermoFisher) supplemented with 10% fetal bovine serum (Sigma-Aldrich) at 37°C in an atmosphere of 5% CO_2_. Cells were sub-cultured three times per week.

#### Cytotoxicity Assay

To estimate the potential cytotoxicity of dendrimers resazurin assay was performed (20) to investigate cells. Cells were seeded into 96-well black plates at a density of 1.5 × 10^4^ cells per well and treated with increasing concentrations of dendrimers for 24 and 72 h. Following the incubation, resazurin was added to the culture medium to a final concentration of 10 μg/ml and the plates were incubated at 37°C in darkness to allow conversion of resazurin to resorufin. Fluorescence of metabolized resazurin in the cell suspension was measured after 30 and 90 min at 530 nm excitation and 590 nm emission using EnVision plate reader (PerkinElmer, Waltham, USA). Cell viability was calculated as the increase in resorufin fluorescence between 30 and 90 min and was presented as percentage of untreated control.

#### Gene Expression Assay

The expression level of NF-κB marker genes (*IGFBP3*, *TNFAIP3*) or cytokine genes (*TNF*, *IL-4*) was determined by quantitative real-time RT-PCR. Aliquots of 1 × 10^6^ of THP-1 cells were cultured for 24 h with the PPI-m DS G4 (or PPI-m DS G3) glycodendrimer. In some experiments, cells were co-incubated with parthenolide (Sigma-Aldrich) as a pharmacological inhibitor of the NF-κB pathway. Following incubation, cells were harvested, washed once with PBS and processed for mRNA using Dynabeads mRNA DIRECT Kit (ThermoFisher) according to manufacturer’s protocol for cultured cells. Complementary DNA (cDNA) was then transcribed from mRNA via High Capacity cDNA Reverse Transcription Kit (ThermoFisher). It was subsequently used for real-time PCR amplification using the Express SYBR GreenER qPCR SuperMix Universal (ThermoFisher) according to manufacturer’s protocol. Each 10 μl reaction volume contained 1 μl of cDNA and 0.4 μM of forward and reverse primers. PCR reactions were performed in 96-well microplates using the CFX96 Real Time PCR Detection System (Bio-Rad). The following intron-spanning gene-specific primers were used: IGFBP3 REV (5′-AGCTCCAGGAAATGCTAGTGAGTC-3′); IGFBP3 FOR (5′-GGTGGAACTTGGGATCAGACAC-3′); TNFAIP3 REV (5′-CTTGACCAGGACTTGGGACTTTGC-3′); TNFAIP3 FOR (5′-AGCCATTGTGCTCTCCAACACCTC-3′); TNF REV (5′-ATCTCTCAGCTCCACGCCATTG-3′); TNF FOR (5′-CCAGGCAGTCAGATCATCTTCTCG-3′); IL4 REV (5′-TCTCATGGTGGCTGTAGAACTGC-3′); IL4 FOR (5′- ACAGCCTCACAGAGCAGAAGAC-3′); as well as primers specific for housekeeping *HPRT1* and *TBP* genes: HPRT1 FOR (5′-TGACACTGGCAAAACAATGCA-3′); HPRT1 REV (5′-GGTCCTTTTCACCAGCAAGCT-3′); TBP FOR (5′-CACGAACCACGGCACTGATT-3′); TBP REV (5′-TTTTCTTGCTGCCAGTCTGGAC-3′). The above reference genes were selected previously for the cell/treatment combination according to the GeNorm procedure (21). The expression level of assayed genes was calculated by the ΔΔCt method as the number of cognate mRNA copies per 1 copy of geometric-averaged mRNA for reference genes.

#### NF-κB Translocation Assay

Cultured THP-1 cells were stimulated for 2.5 h with the PPI-m DS G4 glycodendrimer or with TNFα as a positive control. Aliquots of 5 × 10^4^ cells were then withdrawn from the culture and transferred to a thin-bottom 96-well plate coated previously with poly-lysine. After 10 min of sedimentation at 37°C, cells were centrifuged (5 min, 100×*g*, RT) to enhance cell adhesion to the plate. Following gentle aspiration of culture medium and a single wash with PBS, cells were immediately fixed for 20 min at room temperature with PBS-buffered 2% formaldehyde solution freshly prepared from paraformaldehyde. Subsequently, cells were washed once with blocking buffer (PBS, 1% BSA, 0.1% Triton X-100) and further incubated with the blocking buffer overnight at 4°C. NF-κB was then probed for one hour with DyLight488-conjugated antibody diluted in the blocking buffer (anti-NF-κB p65 DyLight488 rabbit monoclonal antibody, EP2161Y, Abcam, 1:100). Finally, after three wash cycles with the blocking buffer, nuclei were stained with 5 μM Hoechst 33342 dye for 5 min and cells were stored protected from light in PBS/0.02% NaN_3_ at 4°C until imaging. Measurement of NF-κB p65 translocation from cytoplasm to nucleus was conducted with a high-content screening platform (ArrayScanVTi from ThermoFisher) using manufacturer’s proprietary software and protocol for measuring the ratio of nuclear to cytoplasmic staining (22). Measurements were made for >2500 cells per single well, ratios were averaged within the well and taken as single biological replicates. Data is presented as percentage of control (untreated) translocation ratio. The number of replicates indicated in the figure refers to the number of biological replicates of treatment experiment.

#### Electrophoretic Mobility Shift Assay (EMSA)

THP-1 cells (1 × 10^6^) were harvested after 2.5 h of treatment, washed once with PBS and centrifuged at 500×*g* for 3 min. Supernatant was carefully removed, leaving the cell pellet as dry as possible. Nuclear extracts were then prepared using the NE-PER Nuclear and Cytoplasmic Extraction Reagents (ThermoFisher) with the Halt Protease and Phosphatase Inhibitor Cocktail (ThermoFisher) according to the manufacturer’s recommendation. Protein concentration of extracts was determined using the Microplate BCA Protein Assay Kit – Reducing Agent Compatible (ThermoFisher) and aliquots were frozen at −80°C until use.

Nuclear extracts were analyzed for the presence of active (DNA-binding) NF-κB using double-stranded oligonucleotides probes with the NF-κB consensus binding sequence, labeled with IRDye 700 infrared fluorescence dye (5′-AGT TGA GGG GAC TTT CCC AGG C-3′, consensus site is underlined, custom-synthesized by Metabion International AG). Extracts were incubated for 30 min at 4°C with 0.5 μg/ml salmon sperm DNA in binding buffer: 5% glycerol, 10 mM MgC1_2_, 1 mM DTT, 50 mM NaCl, 0.1% NP-40, 0.4 μM ZnCl_2_ and 10 mM Tris-HCI, pH 8 with or without the addition of 2 pmol/μl of the competing, unstained oligonucleotide probe. After this time, labelled NF-κB probes were added to the mixture at the final concentration of 0.02 pmol/μl and further incubated 30 min at 4°C. DNA-protein complexes were analyzed by electrophoresis in denaturing conditions on a 12% polyacrylamide gel at 4°C. The probe-protein complexes were visualized on an Odyssey IR imager (Li-Cor). Band intensities were quantified digitally using ImageJ software.

#### Cytokine Assay

Cultured THP-1 cells were stimulated for 24 h with the PPI-m DS G4 glycodendrimer. Subsequently, cells were removed by centrifugation (5 min, 5000×*g*, RT) and protein concentration of TNFα and IL-4 was measured in the supernatant using Quantikine ELISA kits (R&D Systems). The assay was performed strictly according to manufacturer’s protocol, the absorbance was read in an EnVision plate reader (Perkin Elmer) at 450 nm.

#### Statistics

For statistical significance testing we used one-way ANOVA for concentration series followed by post-hoc Tukey’s test for pairwise difference testing. In all tests, *p* values < 0.05 were considered to be statistically significant. Data are presented as arithmetic mean ± S.E.M.

## Results

**Fig. 1:**
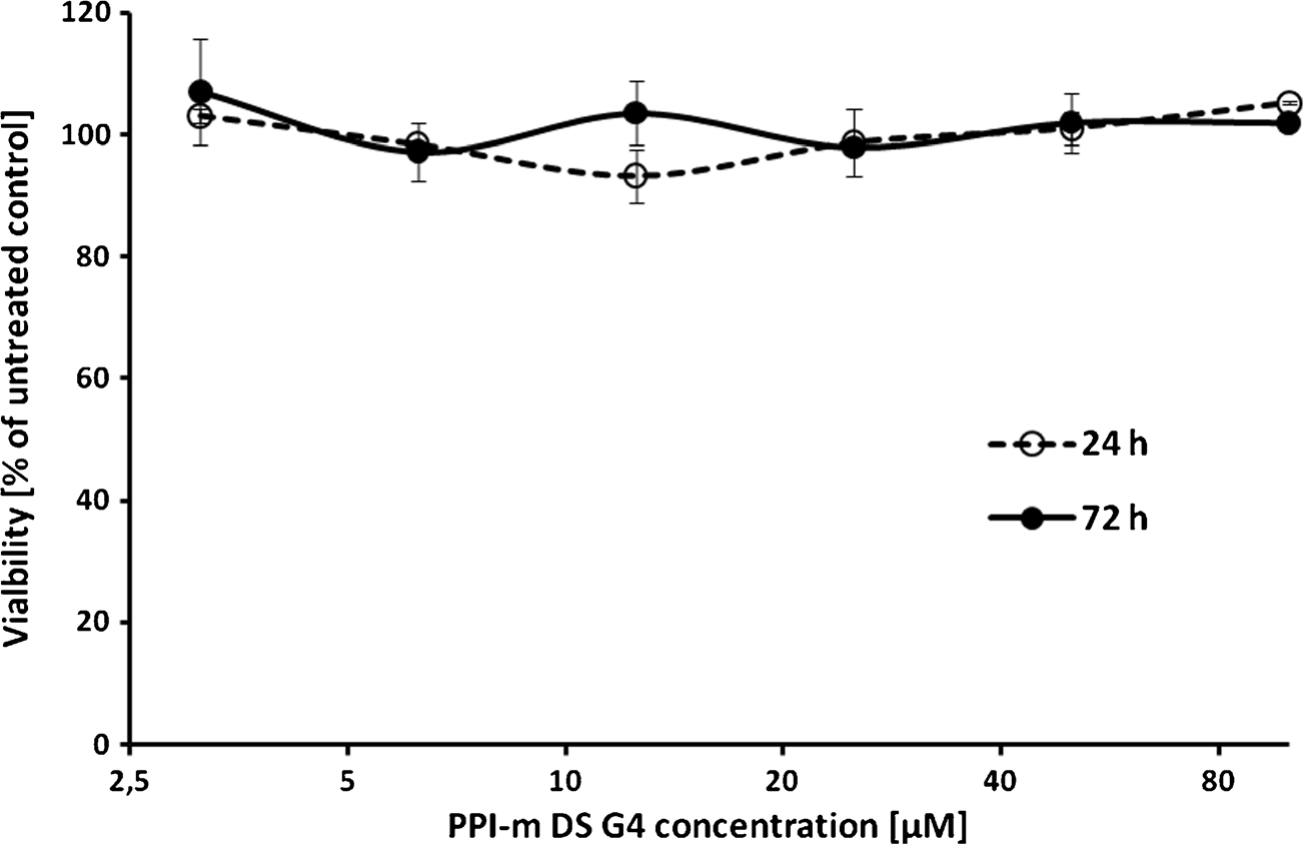
Effect of PPI-m DS G4 dendrimers on the viability of THP-1 cells. Viability was determined by the resazurin assay after 24 h (*empty symbols*) and 72 h (*filled symbols*) of treatment with dendrimers. Data presented as percentage of viability of control (untreated) cells, average ± S.E.M., *n* = 4.

In order to test the biocompatibility of PPI-m DS glycodendrimers with the cellular model applied in this study, the survival rate of THP-1 cells following treatment with a range of PPI-m DS G4 concentrations (3.125–100 μM) was measured using the resazurin assay method. Cells were treated for 24 and 72 h and compared with untreated cells (Fig. 1), demonstrating that at these concentrations PPI-m DS G4 glycodendrimers do not influence cell viability to any measurable extent even during a prolonged incubation.

**Fig. 2:**
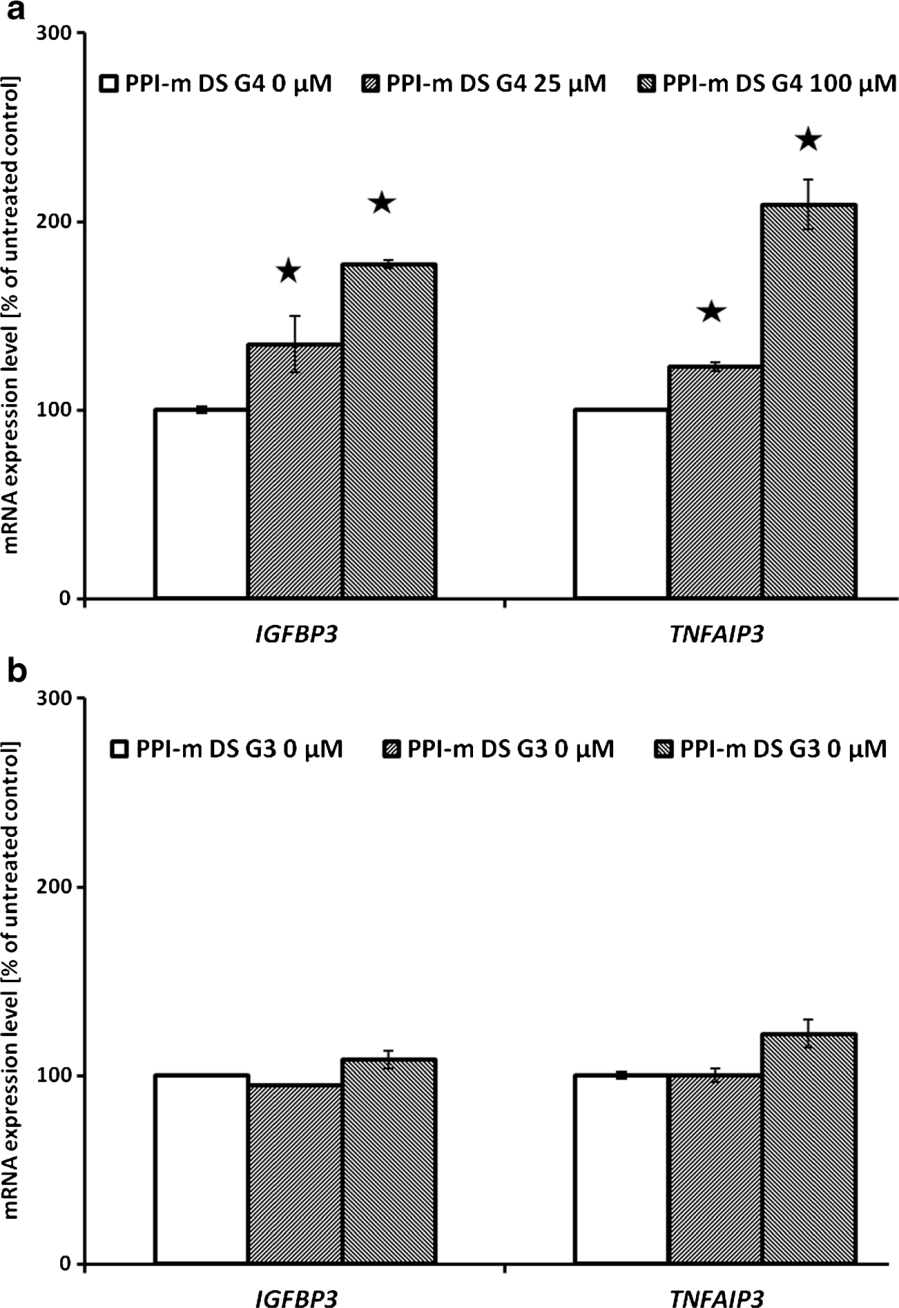
Panel (**a**) and panel (**b**) show the effect of PPI-m DS G3 or PPI-m DS G4 dendrimers, respectively, on mRNA expression of *IGFBP3* and *TNFAIP3* marker genes in THP-1 cells. Gene expression was determined by real-time RT-PCR after 24 h of treatment with dendrimers. Data presented as percentage of cognate mRNA expression in control (untreated) cells (number of cognate mRNA copies per 1 copy of geometric-averaged mRNA for reference genes), average ± S.E.M., *n* = 6.

Since our main goal was to identify potential novel bioactivities of glycodendrimers, which might lead to immunomodulatory side effects by their interference with cellular signaling pathways, we evaluated gene expression at the mRNA level of a set of marker genes. These genes were selected as known targets of main cellular pathways that respond to xenobiotic stress and contribute to immunomodulation and immunotoxicity in myeloid cells: the Jak/STAT pathway (*SOCS3*, *OSM*), the NF-κB pathway (*TNFAIP3*, *IGFBP3*), the Keap1/Nrf2 pathway (*PRDX1*, *SOD2*) and the ER stress pathway (*CHOP*, *EDEM*). In THP-1 cells treated with PPI-m DS G4 glycodendrimers at different concentrations (25 or 100 μM) for 24 h, a statistically significant effect – activation of mRNA expression – was detected only for the two gene markers of the NF-κB pathway (Fig. 2a). Expression levels of all the other marker genes remained constant after treatment (data not shown). In the case of *TNFAIP3* and *IGFBP3*, the induction of expression was dose-dependent, with up to 2-fold increase for the top concentration tested (100 μM). As a control of role of glycodendrimer size, we performed the same experiments using a corresponding lower-generation dendrimer – PPI-m DS G3. At the same concentrations (25 or 100 μM), no statistically significant stimulatory effect on the expression of NF-κB pathway could be detected (Fig. 2b).

**Fig. 3:**
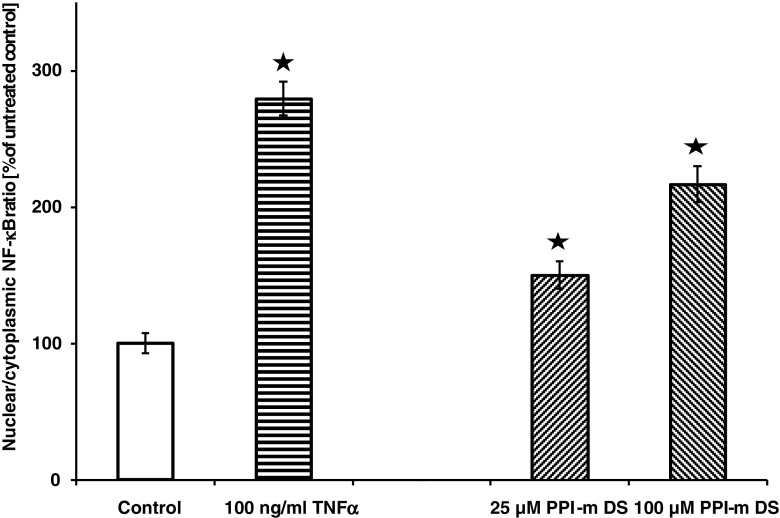
Effect of PPI-m DS G4 dendrimers on nuclear translocation of NF-κB in THP-1 cells. Subcellular localisation of NF-κB was determined by high-content screening (immunofluorescence with anti-p65 antibody and automated microscopy/image analysis) after 2.5 h of treatment with dendrimers. Treatment with 100 ng/ml TNFα was used as positive control of NF-κB nuclear translocation. Data presented as percentage of nuclear to cytoplasmic staining ratio in control (untreated) cells, average ± S.E.M., *n* = 6.

Since activation of marker gene expression is an indirect proof of pathway activation and may be caused by other effects, we subsequently set out to prove the direct involvement of the NF-κB pathway in cellular response to glycodendrimers. Since a hallmark of NF-κB activation is the translocation of this transcription factor from the cytoplasm to the nucleus, we used a high-content screening assay to reliably and objectively quantify this translocation in glycodendrimer-treated cells. Figure 3 shows that treatment with PPI-m DS G4 induces NF-κB translocation in a dose-dependent manner to an extent similar to that observed for marker gene induction (more than 2-fold). In the same setting, a control NF-κB-activating stimulus, TNFα treatment, was able to induce stronger NF-κB translocation than the top glycodendrimer concentration, indicating that the mode of stimulation of the NF-κB pathway by PPI-m DS G4 is probably distinct from specific receptor-mediated interactions.

**Fig. 4:**
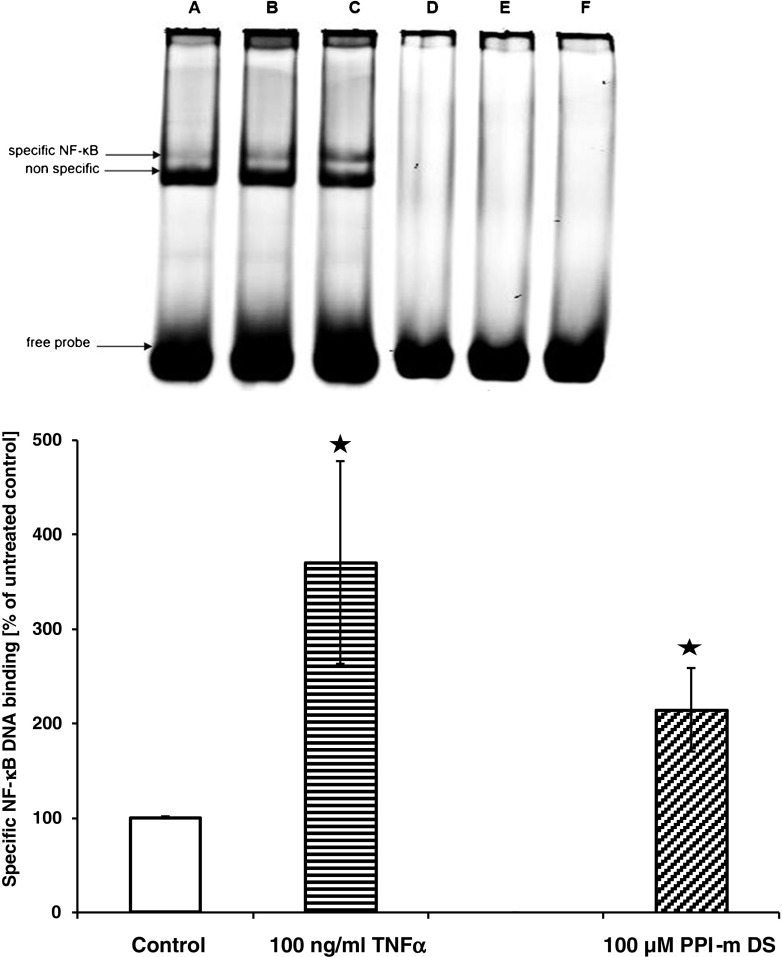
Effect of PPI-m DS G4 dendrimers on DNA-binding activity of nuclear NF-κB in THP-1 cells. Binding of NF-κB to a consensus DNA probe was determined by EMSA (nuclear extract incubated with IRDye 700-labelled probe, imaging on Odyssey IR imager) after 2.5 h of treatment with dendrimers. Treatment with 100 ng/ml of TNFα was used as positive control of NF-κB activation. Panel (**a**) shows a representative EMSA image – the upper shifted band corresponds to specific NF-κB binding, the stronger lower shifted band corresponds to a known non-specific protein interacting with the consensus sequence. The lanes in the image correspond to: *A*, *D* – untreated cells, *B*, *E* – PPI-m DS 100 μM, *C*, *F* – TNFα 100 ng/ml; *A*, *B*, *C* – no probe competition; *D*, *E*, *F* – 100× excess of unlabelled probe. Panel (**b**) shows quantification (by computer image analysis using ImageJ software) of the specific NF-κB-bound band. Data presented as percentage of band intensity in control (untreated) cells, average ± S.E.M., *n* = 3.

The function of NF-κB after translocation into the nucleus depends on its ability to bind specific DNA regulatory elements and influence the transcriptional rate. To confirm that PPI-m DS G4 glycodendrimers can cause this effect, we measured active nuclear NF-κB content using the electrophoretic mobility shift assay (EMSA). Figure 4a shows that PPI-m DS G4 treatment of THP-1 cells promotes stronger binding of a nuclear protein to an NF-κB-specific consensus oligonucleotide, demonstrated as enhanced intensity of a mobility-shifted band. An even stronger enhancement of this band is seen with the positive control stimulus TNFα. Another shifted band of strong intensity appears below this band – this band is not enhanced either by TNFα or PPI-m DS G4 treatment. It has been demonstrated in previous literature using the same probe sequence that this band is due to non-NF-κB nuclear proteins binding to the same sequence and that the weaker upper band corresponds to actual active p65 binding (23). For this reason, we quantified this upper specific band in biological replicate experiments by image analysis (Fig. 4b) – the results are highly similar to those obtained for nuclear translocation and marker gene expression, with ca. 2-fold induction by 100 μM PPI-m DS G4.

**Fig. 5:**
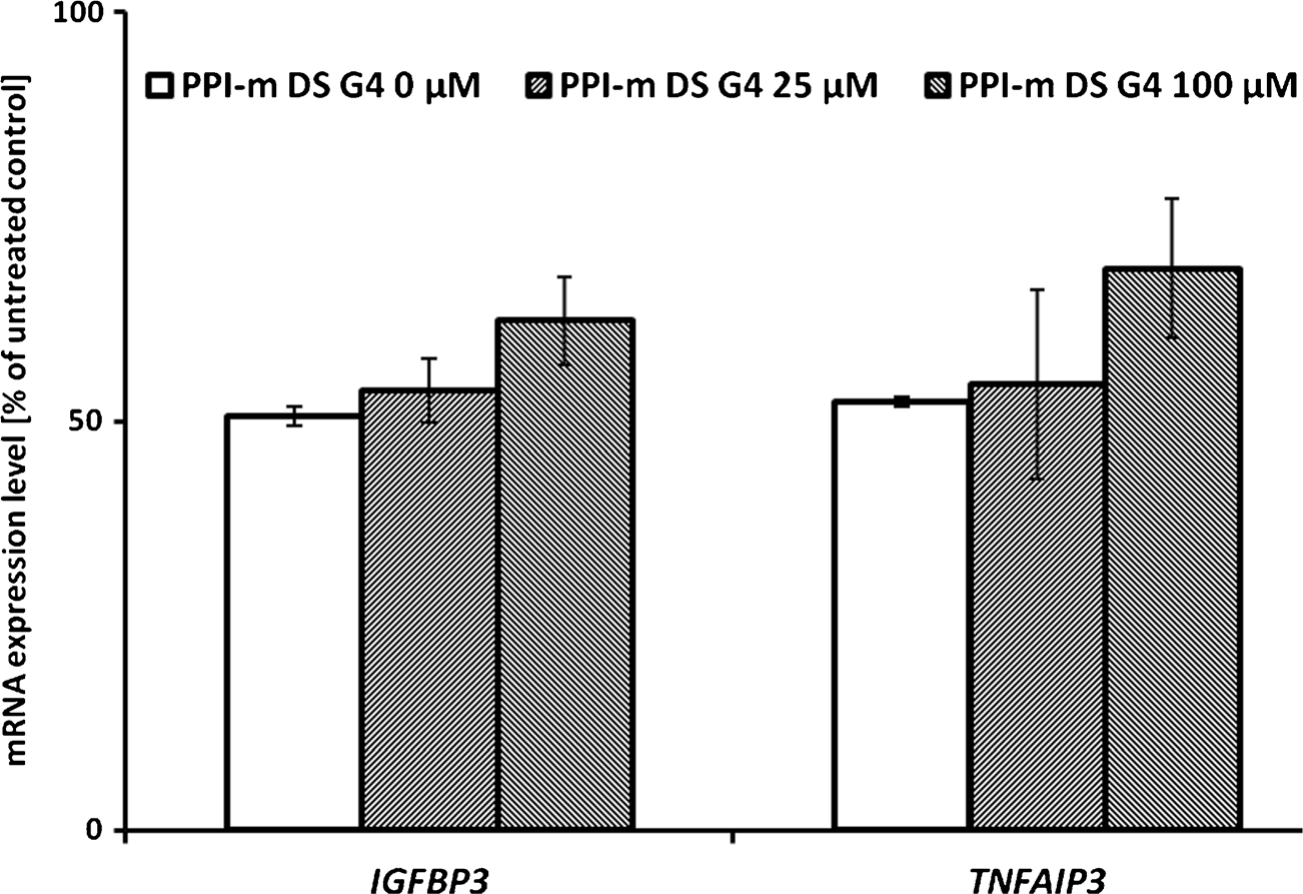
Effect of NF-κB inhibitor parthenolide on the induction of mRNA expression of *IGFBP3* and *TNFAIP3* marker genes by PPI-m DS G4 dendrimers in THP-1 cells. Gene expression was determined by real-time RT-PCR after 24 h of treatment with 5 μM parthenolide and dendrimers. Data presented as percentage of cognate mRNA expression in control (untreated with dendrimers or parthenolide) cells (number of cognate mRNA copies per 1 copy of geometric-averaged mRNA for reference genes), average ± S.E.M., *n* = 4.

Final confirmation of the involvement of classical NF-κB pathway in marker gene induction by glycodendrimer treatment comes from the results of application of parthenolide, which is a known and specific pharmacological inhibitor of this pathway, working by inhibition of I-κB kinase and/or direct modification of the p65 protein (24). As expected, application of 5 μM parthenolide alone caused a ca. 2-fold decrease in basal NF-κB marker gene expression in THP-1 cells (Fig. 5). Significantly, concurrent application of parthenolide prevented the induction of *IGFBP3* and *TNFAIP3* gene expression by PPI-m DS G4 glycodendrimers in the same conditions as those in which the induction was demonstrated previously. This indicates that the induction signal is indeed mediated by the NF-κB pathway.

**Fig. 6:**
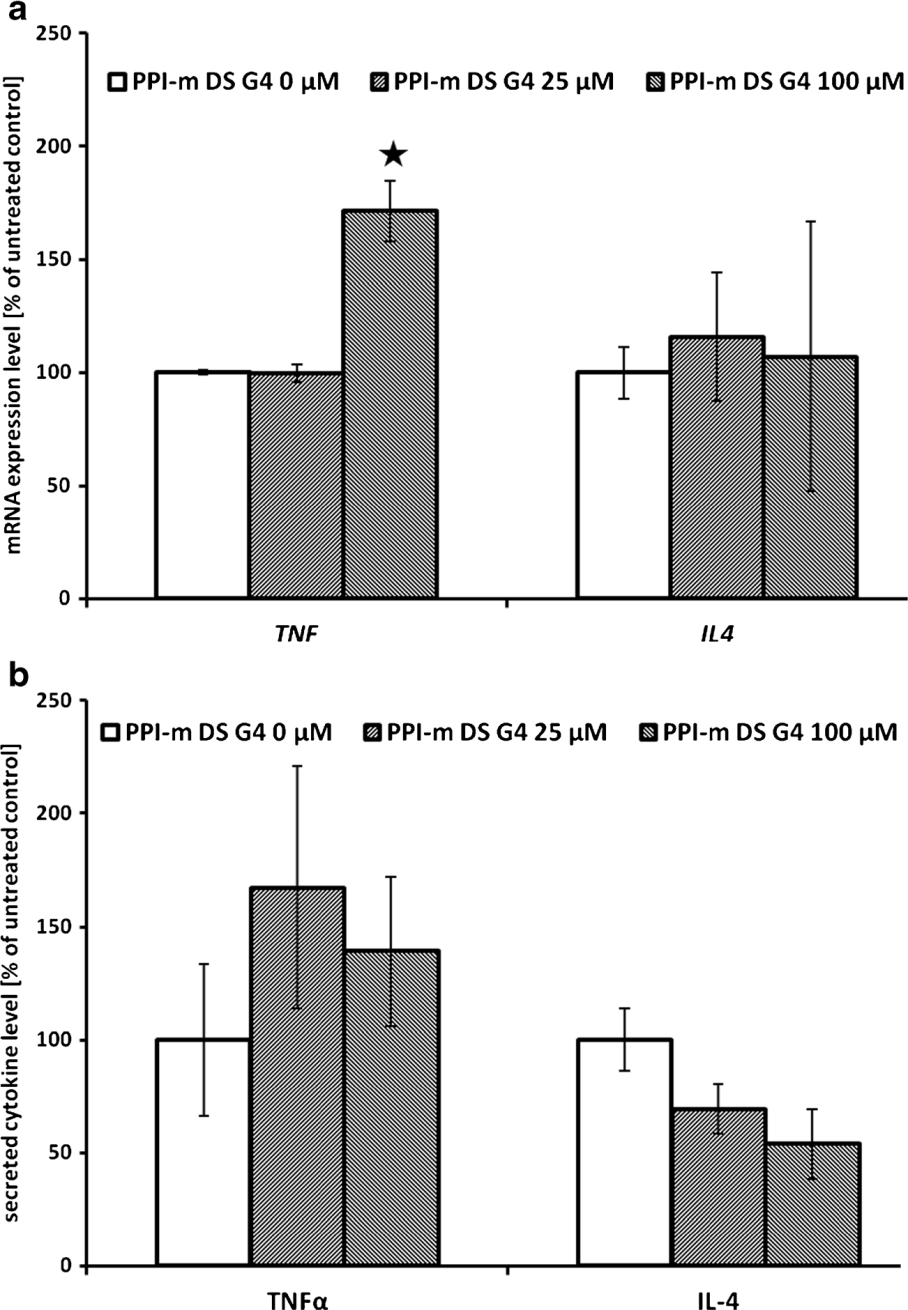
Effect of PPI-m DS G4 dendrimers on mRNA and protein expression of cytokines in THP-1 cells. Panel (**a**) gene expression at the mRNA level was determined for *TNF* and *IL4* genes by real-time RT-PCR after 24 h of treatment with dendrimers. Data presented as percentage of cognate mRNA expression in control (untreated) cells (number of cognate mRNA copies per 1 copy of geometric-averaged mRNA for reference genes), average ± S.E.M., *n* = 3. Panel (**b**) gene expression at the protein level was determined for TNFα and IL-4 by ELISA in cellular supernatants after 24 h of treatment with dendrimers. Data presented as percentage of cytokine levels in supernatants form control (untreated) cells, average ± S.E.M., *n* = 3.

We set out on a preliminary study of physiological relevance of the immunomodulatory potential of PPI-m DS G4 glycodendrimer interference with the NF-κB pathway. We verified the impact of dendrimer treatment on the expression of TNFα (a model pro-inflammatory cytokine induced by NF-κB activation) and IL-4 (a regulatory cytokine which should not be directly influenced by NF-κB activation in myeloid cells). At the level of mRNA expression, verified by real-time RT-PCR, the highest concentration of PPI-m DS G4 (100 μM) significantly induced the expression of the *TNF* gene, while no impact on the expression of the *IL4* gene was detected (Fig. 6a). On the other hand, levels of both secreted cytokines, measured at the protein level by ELISA in cellular supernatants, were not influenced by glycodendrimer treatment (Fig. 6b).

## Discussion

The identification and understanding of molecular mechanisms by which dendritic nanostructures can change the cellular environment provide valuable guidance for strategies using these particles clinically, e.g. as carriers of bioactive compounds (25) or as diagnostic contrast agents (26). The successful use of dendrimers in living systems relies on their biocompatibility, which encompasses their impact on intrinsic homeostasis of the organism and cell. Dendrimers have important features such as low cytoxicity (27), selective capacity to deliver active compounds (28) and degradability in living systems (29). These features are dependent mainly on specific surface composition, including charge (30).

Glycodendrimers, which first appeared in the literature in 1993 (31), are one of the most promising class of dendrimers. They include biologically relevant carbohydrate, oligosaccharide or glycopeptide ligands at the periphery of dendritic scaffolds (PEI - poly(ethylene imine), PAMAM - poly(amidoamine), PPI - poly(propylene imine) and others), making them accessible for binding interactions with multiple biomolecules (32). For example, it has been reported that dendrimers with surface carbohydrate ligands are successfully recognized by lectin receptors in biological systems. A mannose moiety conjugated to the amine terminals of PPI dendrimers was capable of interacting with lectin receptors and become internalized by macrophages (33) and undergo receptor-mediated endocytosis, which provides a mechanism for the selective uptake of specific nanoparticles (34). Thus, immune system-targeted sugar-modified dendrimers are an exciting avenue of research not only in terms of delivering drugs or antigens, but also with regard to direct impact of dendrimers on immune system function and regulation. Use of glycodendrimers combines the advantages of carbohydrate modification for receptor interactions and nanoparticle form for stability, uniformity and defined pharmacokinetics.

In our previous studies, we have concentrated on novel properties of maltose-modified glycodendrimers. They were tested as potential drug delivery agents for nucleotides in ovarian carcinoma cells and liver hepatocellular cells (19). Polyplexes consisting of DNA and PEI with open maltose shell were shown to undergo simultaneously clathrin-dependent and clathrin-independent endocytic processes in ovarian carcinoma cells (35). In PPI dendrimers of the fourth generation, the extent of maltose modification (open shell, with positive charge, or dense shell, with close to neutral charge) had a very strong impact on their cytotoxicity, internalization pathways, intracellular trafficking and distribution in primary and metastatic melanoma cell lines (36). Similarly, PPI-m OS and DS dendrimers differed in cytotoxicity profiles in Chinese hamster ovary and human ovarian carcinoma cells (13) and in their ability to cross blood–brain barrier (12).

In the present study, we used dense shell maltose-modified 4th generation poly(propylene imine) glycodendrimers (PPI-m DS G4), which were previously shown to be significantly more biocompatible (less cytotoxic) that the open shell variant, to verify their potential immunomodulatory function in model cells of the myeloid lineage – the human monocytic cell line THP-1. We wanted to identify and understand potential molecular mechanisms by which glycodendrimers can influence the cellular environment in a non-toxic manner. Thus, we chose the THP-1 cell line since its signaling pathways are well characterized and they are commonly used as models for monocytes or non-activated macrophages. We used non-differentiated THP-1 cells because their responses are more general and do not depend on upregulated receptors (37). Since monocytes are prototypic myeloid cell lines involved in the innate immune response, expressing i.a. lectin receptors, which function as pattern recognition receptors for pathogens (38), we postulated that maltose-modified glycodendrimers may interact with signaling pathways important for cellular function and in that manner potentially exert an immunomodulatory effect.

Because our main purpose was to identify novel immune-related bioactivities of fully maltose-modified dendrimers, we have not included unmodified (or partially-modified, so-called open shell) PPI dendrimers in our study. These dendrimer species were previously demonstrated to be strongly cytotoxic, precluding their application in cellular exposure at concentrations used in this study (12). Moreover, the presence of amino groups on the surface of unmodified or partially modified particles conveys a high affinity for nucleic acids (8), making it impossible to efficiently isolate cellular RNA in their presence for quantitative RT-PCR.

Our results show that PPI-m DS G4 dendrimers indeed activate the NF-κB signaling pathway, which is one of the most important pathways for innate immunity. We demonstrated this effect at several levels: in our experiments, the tested glycodendrimers induced NF-κB nuclear translocation, specific DNA binding activity, activated the transcription of marker genes known to be specific NF-κB targets (*IGFBP3* and *TNFAIP3*), as well as of the *TNF* gene encoding TNFα, a prototypic pro-inflammatory cytokine regulated by NF-κB. These effects were exerted in a dose-dependent manner and dendrimers had similar potency in all applied assays, demonstrating a common molecular mechanism. It is important to observe that the influence of PPI-m DS G4 treatment on THP-1 cells was seen at relatively high dendrimer concentrations (25–100 μM), which is significantly higher than concentrations previously tested for drug delivery applications (12,13). Still, at these concentrations no deleterious effect on cellular viability and growth could be seen even after 72 h of incubation. Moreover, even at these high concentrations PPI-m DS G4 had no effect on the expression levels of marker genes, which are sensitive to even slight variations in activity of other signaling pathways important for cellular function and innate immunity (Jak/STAT, Keap1/Nrf2 and ER stress). This means that these glycodendrimers do not cause a generalized cellular stress (such as oxidative stress or unfolded protein response, both of which are also known to induce NF-κB activity) and their impact on NF-κB activity is specific, potentially receptor-mediated. Thus, the main take-home message from our study is that if maltose-modified dendrimers are to be used at low concentrations for clinical goals such as drug delivery, they should be fully bioorthogonal for the innate immune system; if, on the other hand, they need to be applied at relatively high concentrations, their immunomodulatory potential has to be taken into account despite the generally high biocompatibility they otherwise demonstrate.

It is interesting to note that application of lower-generation (G3) glycodendrimers, which were otherwise chemically equivalent to the PPI-m DS G4 particles used in the rest of the study, did not yield a significant stimulation of NF-κB activity in the gene expression part of the study. The biocompatibility of the smaller nanoparticles has previously been comprehensively described and found to be analogous to the G4 variant (12). Two potential explanations for this advantage of 4th generation glycodendrimers may be proposed: lower dendrimer size may be inconvenient for direct interactions with monocyte pattern recognition receptors on the surface of THP-1 cells, especially if dendrimer internalization (e.g. by phagocytosis) plays a role in this recognition. Alternatively, the carbohydrate load of the smaller molecules (since the whole surface is uniformly modified with maltose molecules, one generation difference in size translates to a 2-fold difference in the amount of surface-accessible moieties) may not be enough to trigger a relevant signaling response.

It is important to note that activation of the NF-κB pathway by even the highest dendrimer concentrations tested was still significantly weaker than activation by TNFα, a cognate ligand for a receptor known to strongly activate the NF-κB pathway and to contribute to the inflammatory response. Moreover, while the top concentration (100 μM) of PPI-m DS G4 did activate TNFα expression at the mRNA level (TNFα is both a target and an activator of the NF-κB pathway), no corresponding increase in active cytokine secretion from the cells was seen at the protein level. This is expected for a specific and limited NF-κB activation event, since TNFα secretion from the cell is a complex process regulated at several NF-κB-independent stages (39). Therefore, the potential effect of PPI-m DS G4 on the immune system *in vivo* would most probably not involve full-scale inflammation and would not be *per se* deleterious for the organism. The potential effect of modest NF-κB activation is rather an adjuvant-like effect, which may be very advantageous in dendrimer application for treatment of tumors. Potentially, these nanoparticles can be successfully used not only as vectors of targeted delivery of drugs to cancer cells, but also as stimulants to augment the anti-tumor attack of the innate immune system. This mild adjuvant-like effect of glycodendrimers was previously mentioned in the study on their use in facilitating anti-tumor vaccine application, but no molecular mechanism was proposed (40).

It is interesting to speculate on the nature and specificity of interactions between maltose moieties on the PPI-m glycodendrimers and receptors on THP-1 cells. Monocytic cells express a number of lectin-type receptors active in innate immunity, and most of them can activate the NF-κB pathway upon ligand binding – however, their cognate ligands are usually saccharides which constitute pathogen-associated molecular patterns, such as mannose, trehalose or beta-glycan. However, their is a growing body of research showing that other simple oligosaccharides, even common food components such as maltose, may interact with these receptors when at high concentrations and/or in a locally dense molecular conformation (as is the case with our glycodendrimers). Human dectin-1 (macrophage beta-glucan receptor) has been shown to bind to maltose-agarose with a similar avidity as to mannan-agarose or chitin (41). Mincle, the macrophage trehalose receptor, binds to maltose-Sepharose to a significant extent, although less tightly than to trehalose-Sepharose (42). Also for the main soluble immune lectin, mannan-binding lectin (MBL), it was shown that high concentrations of glucose and short alpha-glucans can competitively displace its cognate ligands (43). Since THP-1 express both dectin-1 and mincle, and our experiments were performed in the presence of bovine serum (containing MBL), any of these molecules may have participated in the observed NF-κB activation.

Thus, our study confirms the overall high biocompatibility of maltose-modified (G4) glycodendrimers in a novel cellular system (myeloid cell line). On the other hand, it provides for the first time a mechanistic basis for potential additional biological effects of their application, specifically by modulating the innate immune system via activation of the NF-κB pathway. This side effect has to be taken into account in any potential clinical application, but is not necessarily a negative effect – the immunostimulatory potency may be a boon for dendrimers used in anticancer or antiviral settings. Further studies are needed on the specific cellular receptors responsible for interaction with glycodendrimers, especially since carbohydrate ligands are known to trigger immune response in many cases, so a role of the maltose moiety can be postulated. Our study demonstrates comprehensively that it is very important to study novel nanoparticles with potential clinical applications not only for their expected activity, but also for collateral actions and their molecular mechanism.

## Conclusions

Maltose-modified PPI G4 dendrimers are highly promising glyconanoparticles in view of the drastic increase in biocompatibility in comparison to their unmodified congeners. However, for any clinical application, a careful examination of even slight interference with normal homeostasis must be studied at the molecular level, as exemplified by our finding that PPI-m G4 dendrimers in the fully modified (dense shell) variant induce the activity of the NF-κB pathway in a cellular model of monocytes – classical effectors of innate immunity. Our results lead to the direct conclusion that exposure to high concentrations of PPI-m DS G4 enhances the transcriptional activity of NF-κB, leading to a moderate induction of expression of genes controlled by this pathway. This induction is incommensurate with strong activation seen upon pro-inflammatory stimulation and does not itself lead to an increase in secretion of pro-inflammatory cytokines by cells. However, in view of the delicate signalling homeostasis in myeloid cells and potential cross-talk mechanisms with other immunomodulatory stimuli, this is a significant finding that leads both to potential avenues of *in vivo* research into dendrimer application as clinical agents (e.g. adjuvants) and to caveats for systemic dendrimer use as theoretically bioorthogonal drug delivery vehicles.

## Abbreviations

*BSA*: Bovine serum albumin
*CHOP*: DNA damage inducible transcript 3
*DTT*: Dithiothreitol
*EDEM*: ER degradation enhancing alpha-mannosidase like protein 1
*ELISA*: Enzyme-linked immunosorbent assay
*EMSA*: Electrophoretic mobility shift assay
*HPRT1*: Hypoxanthine phosphoribosyltransferase 1
*IGFBP3*: Insulin-like growth factor-binding protein 3
*IL-4*: Interleukin 4
*IRDye 700*: Infrared fluorescent dye, 700 nm
*Jak/STAT*: Janus kinase/Signal transducers and activators of transcription
*Keap1/Nrf2*: Monoacidic inhibitors of the kelch-like ECH-associated protein 1/Nuclear factor erythroid 2-related factor 2
*﻿MBL﻿*: Mannan-binding protein
*NF-κB*: Nuclear factor kappa-light-chain-enhancer of activated B cells
*OSM*: Oncostatin-M
*PAMAM*: Poly(amidoamine) dendrimers
*PEI*: Poly(ethylene imine) dendrimers
*PPI-m DS G3*: Dense shell maltose-modified 3rd generation poly(propylene imine) glycodendrimers
*PPI-m DS G4*: Dense shell maltose-modified 4th generation poly(propylene imine) glycodendrimers
*PRDX1*: Peroxiredoxin 1
*RT-PCR*: Real time polymerase chain reaction
*SOCS3*: Suppressor of cytokine signaling 3
*SOD2*: Superoxide dismutase 2, mitochondrial
*TBP*: TATA box binding protein
*THP-1*: Human monocytic cell line
*TNF*: Tumor necrosis factor
*TNFAIP3*: Tumour necrosis factor alpha-induced protein 3
